# Indigenous guardians as an emerging approach to indigenous environmental governance

**DOI:** 10.1111/cobi.13532

**Published:** 2020-07-17

**Authors:** Graeme Reed, Nicolas D. Brunet, Sheri Longboat, David C. Natcher

**Affiliations:** ^1^ School of Environmental Design and Rural Development University of Guelph 50 Stone Road East Guelph ON N1G 2W1 Canada; ^2^ Department of Agricultural and Resource Economics University of Saskatchewan Room 3D34, Agriculture Building 51 Campus Drive Saskatoon SK S7N 5A8 Canada

**Keywords:** environmental management, indigenous peoples, indigenous rangers, indigenous watchmen, self‐determination, autodeterminación, guardias indígenas, manejo ambiental, pueblos indígenas, vigilantes indígenas

## Abstract

Over the past 3 decades, indigenous guardian programs (also known as indigenous rangers or watchmen) have emerged as an institution for indigenous governments to engage in collaborative environmental governance. Using a systematic review of peer‐reviewed literature for research conducted in Australia, Canada, Aotearoa‐New Zealand, and the United States, we sought to characterize the emergence of indigenous guardians in the literature and explore whether guardian approaches are representative of Indigenous approaches to environmental governance. Using a multistep relevance‐screening method, we reviewed 83 articles published since 1995, that report on, critique, or comment on Indigenous guardians. Our findings indicated that most articles on the topic were published in the last decade (88%), focused on Australia (65%), and were in a social science discipline (53%). The lead author of the majority of articles was an academic, although only half of the articles included an indigenous scholar or member of an indigenous group or organization as a coauthor. Finally, 11 articles were on research of guardian programs that were locally led and only 5 exemplified indigenous governance, based on 2 well‐known community‐based monitoring typologies. Our findings indicate that more research is required to understand the implications of current guardian programs for indigenous self‐determination, particularly when such programs are embedded in a broader western environmental governance structure.

## Abstract 2

**Article impact statement**: Since 2010, the number of peer‐reviewed articles on indigenous guardians has risen substantially, signaling their vital role in conservation.

## Abstract (es)

Guardianes Indígenas como una Estrategia Emergente para la Administración Ambiental Indígena

**Resumen**

Durante las tres décadas más recientes, los programas de guardianes indígenas (también conocidos como guardias o vigilantes indígenas) han emergido como una institución para que las administraciones indígenas participen en la administración ambiental colaborativa. Mediante una revisión sistemática de la literatura revisada por pares de proyectos de investigación realizados en Australia, Canadá, Aotearoa‐Nueva Zelanda y en los Estados Unidos buscamos caracterizar el surgimiento de los guardianes indígenas en la literatura y exploramos si las estrategias de guardianes son representativas de las estrategias indígenas para la administración ambiental. Usamos un método de filtración de relevancia con pasos múltiples para revisar 83 artículos publicados desde 1995; estos artículos reportan, critican o comentan sobre los guardianes indígenas. Nuestros hallazgos indicaron que la mayoría de los artículos dedicados a este tema fue publicada en la última década (88%), está enfocada en Australia (65%) y está dedicada a una disciplina de las ciencias sociales (53%). El autor principal de la mayoría de los artículos fue un académico, aunque sólo la mitad de los artículos incluía a un investigador indígena o a un miembro de un grupo u organización indígena como coautor. Finalmente, once artículos investigaron los programas de guardianes liderados localmente y solamente cinco artículos ejemplifican la administración indígena con base en dos tipologías populares de monitoreo basadas en la comunidad. Nuestros hallazgos indican que se requiere una mayor investigación para entender las implicaciones de los programas de guardianes para la autodeterminación indígena, particularmente cuando dichos programas están arraigados en una estructura de administración ambiental occidental más generalizada.

## Introduction

Over the past several decades, indigenous peoples have used various mechanisms, such as the settlement of comprehensive land claims in Canada and the determination of Native Title in Australia, to reassert their nationhood and authority over ancestral territories (Borrows 2002). In those cases where title to ancestral territories remains unrecognized or contested by the state, Indigenous nations have pursued other political strategies, such as enacting forms of consensus decision making with state authorities and constructive conflicts (Maclean et al. 2015). The strategies chosen by Indigenous nations ultimately depend on the politics of state recognition (Coulthard 2014) and the dynamic institutional settings that govern natural resource management in their respective ancestral territories (Armitage 2005). Irrespective of the exact strategy, Indigenous‐led conservation drives socially just increases in conservation (Artelle et al. 2019), reduces species loss, better protects landscapes (IPBES 2019), and reflects locally relevant stewardship practices embedded in the culture, laws, and history of their given Indigenous nation (Tran et al. 2020). One such strategy is reflected in the emergence of indigenous guardians, also known as indigenous Rangers or Watchmen.

Although the concept of environmental monitoring is not a new practice for indigenous peoples, it has been used increasingly as an Indigenous‐led effort to reassert jurisdiction over their ancestral territories (Kotaska 2013; Zurba et al. 2019). Mainstream environmental monitoring programs have increasingly sought to include indigenous peoples (Thompson et al. 2019), drawing on their knowledge and ways of being (i.e., indigenous knowledge systems) to understand the interactions between the land, animals, water, and air (Bowie 2013; Whyte et al. 2015). Efforts have ranged from the integration of indigenous knowledge systems and science (Bohensky & Maru 2011), to the continuation of indigenous subsistence and cultural activities that include monitoring indicators (Heaslip 2008), to the development of appropriate protocols for data management (Pulsifier et al. 2012). Critics of these programs have, however, noted that there has been inadequate consideration of indigenous nationhood in the design of community‐based monitoring (CBM) programs (Alfred 1999; Coulthard 2014; Wilson et al. 2018; Reed et al. 2020*a*). Rather, indigenous peoples are too often treated as stakeholders who, although they can “bring a wider range of knowledge to understand ecosystem change” (Berkes et al. 2007:145; Reed et al. 2020*a*), lack governing influence in decision making. Despite this, scholars increasingly recognize indigenous community‐based monitoring as an exercise of indigenous self‐determination and jurisdiction (Wilson et al. 2018; Reed et al. 2020*a*). Indigenous guardians, through the strategic reversibility of power (Foucault 1991), exemplify how indigenous resistance can reconstitute power relationships and thus support indigenous governance.

As community‐based environmental stewards, indigenous guardians are responsible for a variety of functions, including design of land and sea management plans (Griffiths & Kinnane 2010); intergenerational knowledge sharing (Peachey 2015); and monitoring activities occurring in their lands and territories (Dehcho First Nations et al. 2016). Recently, there has been an upsurge in attention in the political and academic discourse due to the growing sophistication of indigenous peoples in the communication, marketing, and implementation of indigenous guardian programs to partners, including the state (e.g., Indigenous Leadership Initiative 2019); new federal investments in indigenous guardian programs, such as an additional $102 million over 7 years (2021–2028) to support indigenous rangers across Australia, and a new Indigenous Guardians Pilot Program ($25 million over 4 years) to support the development of indigenous guardian programs in Canada; and growing recognition of indigenous controlled territories, such as those known as Indigenous Protected and Conserved Areas (IPCAs) (ICE Report 2018; Roth & Moola 2018; Zurba et al. 2019). Indigenous‐controlled territories have tangible benefits for conservation, climate mitigation, and indigenous self‐determination (Artelle et al. 2019; IPBES 2019; IPCC 2019; Schuster et al. 2019). As this concept continues to gain traction in community and political discussions, it is a good time to take stock of trends in the indigenous guardian literature.

We examined the evolution of indigenous guardian programs through a systematic review of peer‐reviewed literature on research conducted in Australia, Canada, Aotearoa‐New Zealand, and the United States. We sought to characterize the emergence of indigenous guardians in the literature and explore whether those guardian approaches are representative of indigenous approaches to environmental governance. Using a multistep relevance screening, we reviewed articles published since 1995 that report on, critique, or comment on indigenous guardians. We also considered the origins of indigenous guardian and their contributions to conservation policy and practice.

## Methods

### Indigenous Guardians

Indigenous guardians are community‐based environmental stewards who practice their cultural and traditional teachings on the land. These activities, although varying in design and operation, include responsibilities to monitor activities on their lands and territories (Dehcho First Nations et al. 2016); assist in the design of land and water management planning (Griffiths & Kinnane 2010); support cultural revitalization and intergenerational knowledge sharing (Peachey 2015); and support wildlife and harvest monitoring (Garnett & Sithole 2007). Broadly, an individual guardian program reflects the culture, laws, and history of a given Indigenous nation in how they design, combine, and implement their activities. In Canada, at least 30 programs exist. The best known is the Coastal Guardian Watchmen Network, which has been run by 8 First Nations since 2005 on Haida Gwaii. However, the history and institutional frameworks of settler states have also had important implications for their design and implementation. We focused on 4 countries: Australia, Canada, Aotearoa‐New Zealand, and the United States. These countries, also known as CANZUS, were the only countries to register votes against the adoption of the United Nations Declaration on the Rights of Indigenous Peoples (UN Declaration) (Lightfoot 2016). Their opposition, ironically in the context where federal support (Australia and Canada) has been provided to indigenous guardian programs, was related to land rights, self‐determination, and the minimum standard of free, prior, and informed consent (Lightfoot 2016). For this reason, we believe that each country would illustrate the tensions, and intersections, between indigenous governance and western environmental governance. Each case, including the history of guardian programs, is described in more detail in Supporting Information.

### Search Strategy

We conducted a systematic review of the peer‐reviewed, published literature on indigenous guardians. We drew on the methodologies outlined by Berrang‐Ford et al. (2015), Kouril et al. (2015), and Pullins and Stewart (2006). This involved a thorough analysis of guardian‐related literature through the use of a restriction of parameters and materials in the search terms (Petticrew & Roberts 2006). To guide our review, and selection of the appropriate analysis of the trends and gaps, we asked the following questions based on our original objectives: How is the emergence of indigenous guardians represented in the literature? Are guardian approaches discussed in the literature representative of indigenous approaches to environmental governance?

The literature search was finalized on 5 April 2019. We searched 5 databases: EbscoHost (GreenFILE), Web of Science (Core Collection), ProQuest (Agricultural and Environmental Sciences and International Bibliography of the Social Sciences), Engineering Village (Geobase), and CAB Direct. We used these databases to cover the breadth of peer‐reviewed literature that indigenous guardians could be implicated in, such as agriculture, biology, natural resource management, sociology, policy, and environment. Search restrictions were placed on the language (English only), location (Canada, Aotearoa‐New Zealand, Australia, and the United States), and period (1995–2019, reflecting the creation of the first Caring for Country program in Australia). Using the Boolean logic operators “AND” and “OR,” only source types from target literature were retained. The keywords reflected the diverse indigenous peoples in all 4 countries and the various synonyms for indigenous guardians (Table 1).

**Table 1:** Search terms for systematic review of peer‐reviewed literature on indigenous guardians

Main terms	Expanded terms
Guardians	(*steward* ^*^ OR guardian^*^ OR kaitiakitanga^*^ OR watch^*^ OR ranger^*^ OR community‐led OR monitor^*^)
Indigenous	(Aborigine^*^ OR Aboriginal^*^ OR “Torres Strait” OR Māori OR American Indian^*^ OR North American Indian^*^ OR Indian^*^ OR Alaska^*^ Nativ^*^ OR Native Hawaiian OR Hawaii Nativ^*^ OR Native American OR Inuit OR Aleut OR Metis OR First Nation^*^ OR Indigenous)^†^

† Source for indigenous search parameters: https://www.ccnsa-nccah.ca/docs/context/RPT-ReviewResearchDesigns-Saini-EN.pdf.

### Citation Management and Screening Approach

All citations were imported into the software DistillerSR (Evidence Partners, Ottawa) in which G.R. and N.D.B. collaborated on a multistep relevance screening. First, duplicate citations were removed using the DistillerSR duplicate removal function, and then confirmed by G.R.. Second, titles were screened using a review of the titles and abstracts based on the following inclusion criteria: articles refer to indigenous guardians or community‐based and led programs; articles are related to the management, conservation, monitoring, and governance of ecosystems, resources, and species; studies on which articles are based were conducted in Canada, Aotearoa‐New Zealand, Australia, or the United States.

Third, all articles that appeared applicable went through a full‐text review with an analytical framework devised by G.R. and N.D.B.. To test for reviewer bias, we (G.R. and N.D.B.) independently reviewed the full text of 5 articles, confirmed results, and met throughout the screening process to discuss relevant uncertainties.

### Data Extraction and Analytical Framework

**Figure 1:**
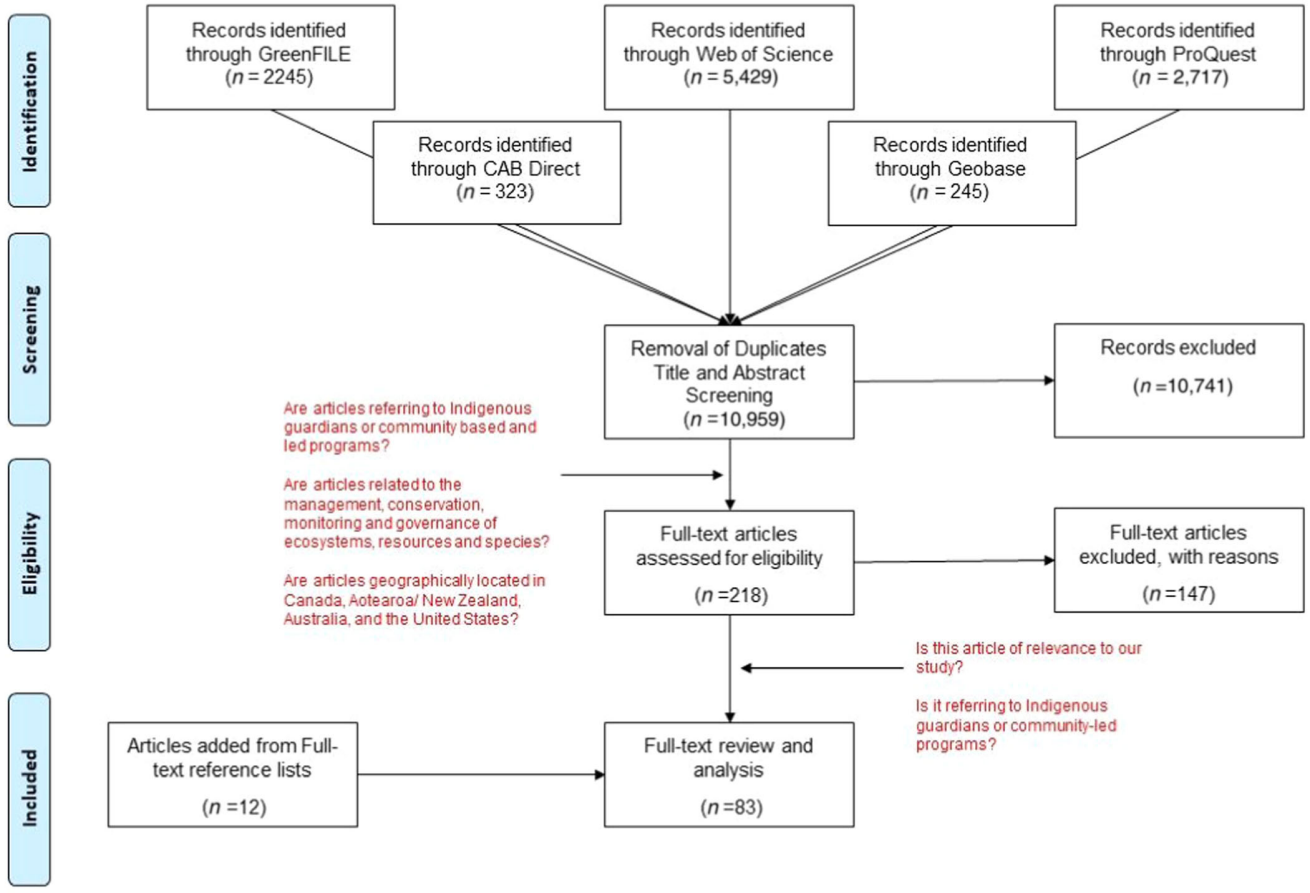
Flow chart of the selection of studies that explored indigenous guardians.

Using the DistillerSR program, we created an analytical framework (with Levels and Forms) to capture both quantitative and qualitative data related to the research questions (Table 2). The type of descriptive information extracted from the full‐text selections included country where the study was conducted, publication year, discipline of study, primary author affiliation, and whether any authors were indigenous people, organizations, governments, etc. (analytical framework inspired by Brunet et al. 2014). To determine indigenous participation, we used a multistep process. First, we examined each author's affiliation and organization. When affiliation was unclear, we sought information on authors’ biographies. Second, we counted whether the opportunities and cobenefits of indigenous guardian programs were explicitly mentioned in the abstract and whether the economics (e.g., cost, distribution of benefits, financial sustainability, and reliance on exogenous actors) were considered. Third, we counted the type of barriers mentioned in the abstract, aiming to determine how certain authors articulated their concerns with the success of indigenous guardian programs. Fourth, for more specific detail on indigenous guardians and their relation to CBM, we created specific questions on how each article related to the typology proposed by Danielsen et al. 2009 (ranking local participation in monitoring programs from externally driven to autonomously driven) and Wilson et al. 2018 (ranking indigenous engagement in CBM from settler governance to indigenous governance) based on approaches from other CBM‐related systematic reviews (Table 2) (Lam et al. 2019). Finally, for those full‐text articles (*n* = 24) identified as worth a second complete reading, we reviewed them with a content analysis (focusing on the themes outlined in the count data; i.e., opportunities and cobenefits, economics and financial sustainability, and barriers) and emerging themes focused on indigenous governance and knowledge; ontological conflict; and a linking or brokering role for indigenous guardians. Figure 1 provides a visual representation of the article‐selection process.

**Table 2:** Framework for analysis of peer‐reviewed articles (*n* = 83) related to indigenous guardians

Study characterization	Count data (yes, no)	Community‐based monitoring typologies
Country of publication Publication Year Discipline of study (social sciences or humanities, natural sciences, life sciences, physical science, interdisciplinary, indigenous) Authorship as represented by the primary author affiliation (university, private organization, nongovernmental organization, federal government, indigenous organization, or government)	At least 1 author represented an indigenous group, program, or community Abstract highlights opportunities of guardian or community‐led programs for indigenous peoples Abstract highlights economics of guardian and community‐based programs Abstract highlights barriers for indigenous peoples associated with guardian and community‐based programs	Type of program, case, management, stewardship, or governance strategy is described in the article according to the Danielsen et al. (2009) typology: externally driven, professionally executed; externally driven with local data collectors; collaborative monitoring with external data interpretation; collaborative monitoring with local data interpretation; or autonomous local monitoring Type of program, case, management, stewardship, or governance strategy is described in the article according to the Wilson et al. (2018) typology: settler governance; settler‐driven cogovernance; indigenous‐driven cogovernance; or indigenous governance

## Results

A total of 10,959 were identified through the initial search. Following the removal of duplicates and articles based on a title screening, 218 articles were selected for an abstract review. Using the criteria outlined above, additional texts were removed and several articles based on a reference review (*n* = 12) were added, leaving 83 articles for full‐text review and analysis.

### Geographic Area of Studies

The majority of studies were conducted in Australia (*n* = 54), followed by Canada (*n* = 13), Aotearoa‐New Zealand (*n* = 10), and the United States (*n* = 2). Several studies were conducted in multiple countries (*n* = 4). Although the Australian Caring for Country was launched in 1995, there were few articles published on it before 2009 (*n* = 10). From 2010 onward, the number of publications grew exponentially, almost tripling their previous rate of production (*n* = 73), and the majority were conducted in Australia (*n* = 46) and Canada (*n* = 11).

### Journals Representation

**Figure 2:**
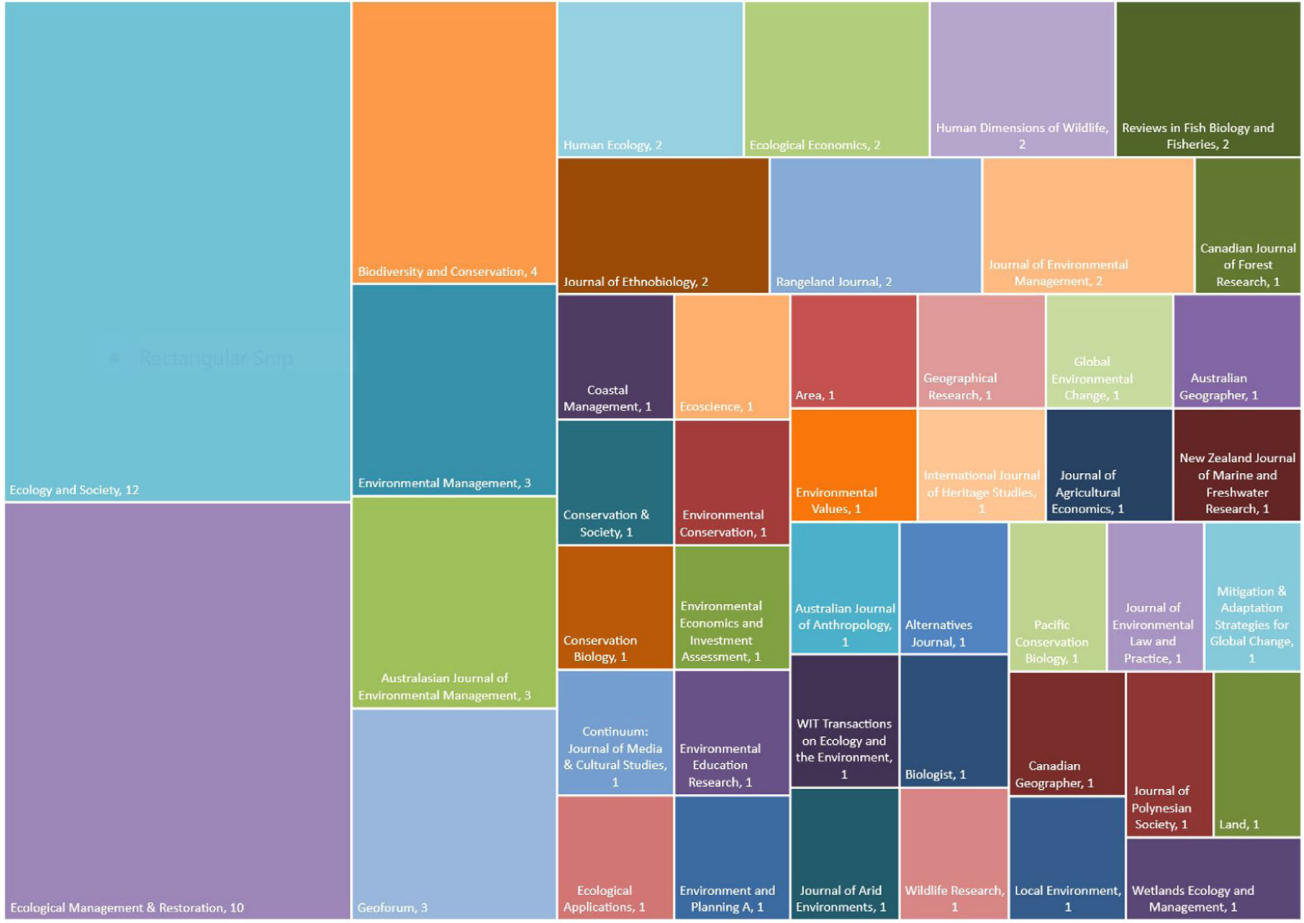
Distribution of journals (n = 46) in which reviewed articles (n = 83) on indigenous guardians were published. Numbers in the squares represent the number of reviewed articles in each respective journal.

The greatest number of articles was published in *Ecology and Society* (*n* = 12), *Ecological Management and Restoration* (*n* = 10), and *Biodiversity and Conservation* (*n* = 4) (Fig. 2). Several journals had 3 articles (*Australasian Journal of Environmental Management, Environmental Management*, and *Geoforum*), but most had only 1 or 2 articles. Fifty‐three percent (*n* = 44) of articles were based in the social sciences and humanities, followed by disciplines characterized as interdisciplinary (30%, *n* = 25) and indigenous (10%, *n* = 8). Only 6% of articles were from the natural sciences, and 1% of articles were from the physical and life sciences, respectively.

### Primary Authorship and Opportunities and Barriers

The majority of articles (*n* = 68) were led by university researchers, followed by national governments (*n* = 4), nongovernmental organizations (*n* = 4), and Indigenous organizations or governments (*n* = 3). The percentage of articles that were led by university researchers (*n* = 68) was higher in Australia (87%) and Canada (85%) than in New Zealand (50%). Over half of the articles included an author who represented an indigenous program, community, or group (*n* = 43). Those that were primarily authored by an indigenous organization or government were all on research conducted in New Zealand (*n* = 2) and Australia (*n* = 1). In abstracts, opportunities and barriers were discussed in 63 and 45 articles, respectively, whereas economics and financial considerations were discussed less (24 articles).

### Indigenous Participation in Guardian Programs

Relative to Danielsen et al.’s (2009) typology, we found that there were clear variations in the level of indigenous participation in indigenous guardian programs. Most articles referred to some level of collaborative monitoring, including programs with external interpretation (*n* = 31) and collaborative data interpretation (*n* = 25). This was followed by studies characterized by externally driven data collection (*n* = 13). The fewest number of programs were autonomous and locally led (*n* = 11), and only 1 study had no local involvement (*n* = 1). Those that were locally led occurred most often in Canada (*n* = 3) and Australia (*n* = 3), followed closely by Aotearoa‐New Zealand (*n* = 2) and multiple countries (*n* = 2). This is consistent with other CBM‐related systematic reviews

Relative to Wilson et al.’s (2018) typology (adapted from Hill et al. 2012), we found that most guardian programs referenced cogovernance arrangements. Forty‐four were settler driven, and 24 were indigenous driven. In total, only 4 (5%) studies presented examples of indigenous governance. These 4 studies were conducted in Aotearoa‐New Zealand (*n* = 2), United States (*n* = 1), and Canada (*n* = 1).

## Discussion

### Emergence of Indigenous Guardians in the Literature

The growth of publications related to indigenous guardians is on the rise since the first Caring for Country unit was created in 1995—aligning well with other studies related to CBM (Kouril et al. 2015; Lam et al. 2019). The majority of articles were based in Australia, most likely a result of the history and recognition of the Working on Country program. Given the relative infancy of the federally funded program in Canada (2018), it is possible that publications based in Canada will increase within the next 5–10 years. In both Aotearoa‐New Zealand and the United States, there was a clear dearth of literature of indigenous guardian programs, likely due to the lack of a formal, federally funded guardian or ranger program.

Only half of the articles led by university researchers included an indigenous person, group, or community as a coauthor. Although this is disappointing, it highlights a genuine concern that nonindigenous scholars may have with misrepresenting the other when engaging with indigenous peoples. However, Shaw et al. (2006) suspect that there is also a dimension of “the politics of indifference.” This hesitancy to engage with indigenous peoples (and their knowledge systems) may entrench colonial modes of knowledge production (Blaser 2013). As a result, scholars call on those researchers working with indigenous peoples to respect and engage with their unique ontologies, including through the development of codesigned and collaborative projects (Ens et al. 2016; Austin et al. 2019). Efforts to cultivate respectful relationships in the research process (Brunet et al. 2016) and to coproduce scalable “two‐ways” indicators for managing indigenous country and conservation must be central to codesigned research (Austin et al. 2018). One promising example of a two‐ways approach is known in Warlpiri (language of Warlpiri people, Australia) as *jarnku mirni mirni*; that is, “… Indigenous and non‐Indigenous people equally and actively sharing their different, yet often complementary, knowledge systems and skill sets towards a joint goal” (Preuss & Dixon 2012:3). Another, presented by Austin el al. (2019), explores how indigenous‐led approaches to maintain the health of Saltwater Country used a regional‐scale collaboration between indigenous knowledge systems, local knowledge systems, and western science. Still, few studies offered tangible pathways to overcome persistent challenges with the integration of knowledge systems, which have been well documented in the literature on CBM (Lam et al. 2019; Reed et al. 2020*b*).

Nonetheless, many articles emphasized the opportunities, or cobenefits, of indigenous guardian programs for indigenous‐led outcomes, such as addressing intergenerational trauma, language, and culture (Holmes & Jampijinpa 2013; Muller 2014); improving health outcomes and clinical indicators (Mackie & Meacheam 2016); and supporting indigenous presence and use of country (Pyke et al. 2018). One article from the United States, for example, speaks to the process of land reclamation as an act of healing: “…to reclaim stolen lands are not solely political projects…but means for healing intergenerational trauma” (Carroll 2014:38). Emphasizing the role of intergenerational knowledge exchange, Sherman et al. (2010) explored how working with youth on the Pine Creek Indian Reservation offers “…hope for future generations of Lakota people to re‐establish their relationships with local reservation ecology” (p. 507). Such reflections are useful to inform not only how the benefits of indigenous guardians are framed (i.e., more than just an economic benefit), but also to articulate the various codependencies that could be answered by these programs and the support for indigenous‐led outcomes. More research is required to explore how best to capture such cobenefits in the evaluation of guardian programs (Bach et al. 2019).

Within the reviewed articles, there was frequent discussion on the economics and financial considerations of indigenous guardians. Major themes included the economic benefits and employment options that indigenous guardians provide (Preuss & Dixon 2012; Mackie & Meacheam 2016) and the chronic underfunding of programs and the challenge of financial sustainability (Fache 2014; Austin et al. 2018). Often, these 2 themes related to one another in contradictory ways. For example, the original purpose of the Working on Country program in Australia was to improve indigenous welfare, rather than protect the environment. Quite paradoxically, however, guardian programs are often lacking the economic arrangements to ensure indigenous welfare, resulting in “…narrowly defined, short‐term, piecemeal, non‐investment oriented, cross‐agency funding” (Woodward 2008:248). In this situation, the provision of financial, institutional, and political resources reinforces an imbalance of power that perpetuates the politics of recognition, whereby Australia, in this case, uses the provision of funds to sustain systems of domination (Alfred 1999) and contributes to the reproduction of the “…very configurations of colonial power that Indigenous Peoples’ demands for recognition have historically sought to transcend” (Coulthard 2014:52). On the Pine Ridge Indian Reservation, structural barriers at the tribal, state, and federal levels prevent the establishment of community‐based institutions (Sherman et al. 2010). The Indigenous Guardians Pilot Project in Canada has not been reviewed; however, early indications are that the funding announced ($25 million over 4 years) is insufficient for the guardian's long‐term financial sustainability (ILI 2019).

As a result of underfunding and structural barriers to revenue generation, indigenous guardians are required to actively fundraise with exogenous funding partners, either universities or institutional investors (Austin et al. 2018). This role, sometimes referred to as a broker, plays an important part in community‐based conservation success, particularly in those situations where “…local knowledge is based on a different epistemology and worldview to government science” (Berkes 2009:5). Many reviewed articles discussed the role that guardians play as mediators between their community and the multitude of actors involved in environmental stewardship (Sherman et al. 2010; Fache 2014; Muller 2014). There were 2 unique tensions discussed in the articles from Australia worth mentioning: overreliance on an individual coordinator for linkages to external funders (Woodward 2008) and the growing tension between guardians and the indigenous nation, landowners, and the broader local community as the state increasingly relies on the guardians for funding arrangements (Fache 2014; Fache & Moizo 2015). Future research in other contexts would add to understanding of whether these tensions are unique to indigenous rangers in Australia (cf. indigenous guardians).

Tensions also manifest with regard to the approaches to environmental management commonly used by indigenous guardians. Carroll (2014) used the emergence of tribal parks to discuss how the maintenance of guardian (or other stewardship‐related programs) requires “…reconciling resource control with traditional teachings that seek to uphold unique tribal relationships with the land and all life” (p. 37). This reality was echoed by several articles describing the context of fire management in Australia (Bach & Larson 2017; Bach et al. 2019), where western approaches to fire ecology have created a social tension between the Ngukurr ranger group community members and Elders. One such example was captured by Fache and Moizo (2015) in their description of an interaction between a young ranger and an older female ranger: “…the male ranger [said], ‘If we don't burn, we won't get the money for this fire project.’ [The older woman replied] ‘You're worried about money; I'm worried for the country’ ” (p. 174). Associating continued financial support with certain practices within these programs can reveal, reproduce, or create power relationships at the expense of indigenous peoples, contributing to the “…invisibility of power of dominant cultures” (Muller 2014:64). The recent implementation of indigenous guardian programs in Canada, for instance, can likely avoid such pitfalls because they reprioritize management approaches to value indigenous‐led cobenefits. Our review results indicate that financial autonomy and sustainability are an essential step in this journey.

### Guardians as Representative of Indigenous Environmental Governance

Exploring whether guardian approaches are representative of indigenous approaches to environmental governance offers an interesting conundrum. Indigenous environmental governance is an emerging discipline that could be captured within a continuum of “resource‐based” and “relationship‐based practices” (Carroll 2014). This is captured in the literature on environmental governance where indigenous peoples are often relegated to stakeholders or participants in decisions pertaining to environmental matters (Reed et al. 2020*a*). In an indigenous governance context, the recognition of indigenous nationhood and self‐determination is paramount (Wilson et al. 2018). Therefore, when considering the objective, one must first ask, what is the dominant policy paradigm that indigenous guardian programs are operating under*?*

In the contexts of formally established guardian programs (such as those in Canada and Australia) and tribal‐based efforts for community‐based institutional development, the dominant policy paradigm, and thus barriers, remains in the control of the state government, creating a sort of “colonial entanglement” (Dennison 2012) for those participating indigenous nations. Even in Aotearoa‐New Zealand, where the Treaty of Waitangi, and thus the concept of *kaitiakitanga*, is embedded in federal legislation such as the Resource Management Act (Morad & Jay 2000), there are still limited examples of where Māori have been given equitable or primary responsibility for the environment (Taiepa et al. 1997; Newman & Moller 2005). In all circumstances, we stress though that indigenous participation is generally better than the alternative to avoid programs or stewardship efforts becoming a prescribed or “deep colonising” management approach (Rose 1995). Indigenous guardians, and the nations they are supporting, are not only balancing these dual and often conflicting roles, but they are also using all opportunities to advance their rights and jurisdiction with or without state recognition (Rist et al. 2019; Reed et al. 2020*a*). For example, the Girringun Aboriginal Corporation has evolved from modest beginnings (i.e., limited statutory indigenous rights) to scaffold comanagement arrangements and joint ventures to “compensate for the absence of clear and strong statutory indigenous rights” (Zurba et al. 2019:1141). Due to the strategic reversibility of power (Foucault 1991), guardian programs can empower indigenous resistance to reconstitute power relationships and thus support indigenous governance (Wilson et al. 2018).

This reality is consistent with the tension articulated in history of the designation of the names *CANZUS* (Australia, Canada, Aotearoa‐New Zealand, and the United States). As the only countries to register votes against the UN Declaration, often due to the supposed uncertainty with the language around free, prior, and informed consent (Lightfoot 2016), it is not surprising that indigenous guardian programs are often caught in a cycle of colonial entanglement (Dennison 2012). Often the environmental management regimes simultaneously require indigenous participants to “resist and contribute at the same time to the proliferation of bureaucracy…” (Fache 2014:283). These types of systems, or the politics of recognition (Coulthard 2014), use recognition (or settler cogovernance) as a tool to sustain systems of domination over indigenous peoples (Alfred 1999), instead of providing greater indigenous authority and self‐determination over ancestral lands. Muller (2014) articulates this tension well: “…until there is a ‘space’ created for Yolngu self‐determination, that is resourced and institutionally acknowledged (rather than operating in the margins of funding contracts) then self‐determination will always be forced into a prescribed, predetermined context” (p. 139).

In Australia, for example, one Ngukurr leader captured this predetermined context well, describing the ranger program as a “new system introduced by the government” (Fache 2014:282), serving to “…extend state power into the very communities that it is supposedly empowering” (Fache 2014:282). In such light, indigenous guardian programs—particularly those in Australia and Canada—must be analyzed under a critical lens to understand whether or not they are preventing, or supporting, sustainable self‐determination (Corntassel 2008). This supports further consideration for the reconciling of indigenous governance arrangements with colonial governance arrangements (Alfred 1999; Hunt 2010; Hill et al. 2012). Future research should explore indigenous guardian literature in non‐CANZUS states to determine their presence, emergence, and results.

### Path Forward

The emerging literature of IPCAs (also known as tribal parks) is indicative of a growing movement to address the colonial history of environmental governance, park and conservation area creation, and the dispossession of land from indigenous peoples (Carroll 2014; Rist et al. 2019; Zurba et al. 2019; Tran et al. 2020). The model of IPCAs challenges the “fortress conservation” model (Domínguez & Luoma 2020) and provides a framework for “…evolved conservation…by exemplifying time‐honoured ways of interacting with environments that support people and places alike” (Artelle et al. 2019:8). In Canada, the recent report produced by the Indigenous Circle of Experts (2018), as part of the domestic work on fulfilling Target 1 of the Aichi Targets, We Rise Together, captures this evolution in defining indigenous‐led IPCAs, where indigenous governments “… have the primary role in determining the objectives, boundaries, management plans and governance structures for IPCAs as part of their exercise of self‐determination” (Indigenous Circle of Experts 2018:36). This approach could provide a “…means for local Indigenous Peoples to re‐assert control over Country that was disrupted by settler colonialization, by reinstituting traditional custodial and cultural responsibilities and building livelihoods based on natural and cultural resources” (Austin et al. 2018:374). As such, we believe that future research should examine the intersection of indigenous guardian programs and the growing emergence of IPCAs, as an approach to reassert indigenous governance over land, resources, and territory (Moola & Roth 2018; Rist et al. 2019; Tran et al. 2020). Clearly, however, our review points to a systematic lack of local indigenous control over the funding and in some cases the design and implementation of indigenous guardian programs.

Further understanding of indigenous guardians specifically—and its linkages to indigenous‐led conservation more generally—would also benefit from studies that review literature broader than just peer reviewed, such as our own. We recognize the methodological limitations implicit within the present study, particularly in an emerging field characterized by indigenous leadership. Indeed, we echo the calls of Ban et al. (2018) and Artelle et al. (2019) to decenter research away from the academy and toward those indigenous leaders on the ground. We hope our review can catalyze future research on indigenous guardians, particularly as these programs solicit increasing attention in Canada and Australia, in a culturally appropriate, respectful, and collaborative manner.

Although academic coverage heralds indigenous guardians approaches as possible pathways to addressing numerous environmental crises, our study revealed that further efforts are needed to understand the implications of guardian programs for indigenous self‐determination as well as indigenous decision‐making institutions and knowledge systems when embedded within broader western environmental governance structures. In particular, we suggest that governments use their acceptance of the UN Declaration and the minimum standard of free, prior, and informed consent to catalyze domestic conversations intended to decolonize conservation policy and practice (Tuck & Yang 2012: Domínguez & Luoma 2020). In doing this, we see great opportunity for current conversations at the Convention on Biological Diversity and the Post‐2020 framework to highlight the contributions and leadership of indigenous‐led conservation and, by extension, indigenous guardians. Although few functional solutions to these complex issues are currently proposed in the literature, we are confident that engaging with indigenous governance is fundamental to achieving conservation and climate targets.

## Supporting information

A history of guardian programs in CANZUS countries (Appendix S1), a list of the full‐text reviewed articles (Appendix S2), and the authors’ biographies and affiliations (Appendix S3) are available online. The authors are solely responsible for the content and functionality of these materials. Queries (other than absence of the material) should be directed to the corresponding author.

Supplementary Material

Supplementary Material
