# Supplementary material for: Indigenous guardians as an emerging approach to indigenous environmental governance
Source: Conserv Biol. 2020 Jul 17;35(1):179–89. doi: 10.1111/cobi.13532 (PMC7984387; doi:10.1111/cobi.13532)
Supplement: Supplementary file 1 — A history of guardian programs in CANZUS countries (Appendix S1), a list of the full‐text reviewed articles (Appendix S2), and the authors’ biographies and affiliations (Appendix S3) are available online. The authors are solely responsible for the content and functionality of these materials. Queries (other than absence of the material) should be directed to the corresponding author. [file COBI-35-179-s002.docx]

### Appendix S1: A history of Guardian programs in CANZUS countries

### Australia & Aotearoa / New Zealand

The histories of Indigenous Peoples now located in Australia and Aotearoa / New Zealand cannot be more distinct. While it is out of the scope of this paper to discuss in detail the differences, suffice it to say that their unique histories have significant implications for how land governance and resource management occurs, thus influencing the role and evolution of Indigenous Guardians.

In Aotearoa, the principles of the Treaty of Waitangi (1840) guide the relationship around resource management, including in federal pieces of legislation such as the Resource Management Act (Morad & Jay 2000). One of the concepts embedded into these is that of *kaitiakitanga* - literal translation is *Guardianship* - but at its most fundamental level represents a system of Maori beliefs that encompass the spiritual, environmental, and human spheres (Kahui & Richards, 2014). According to Kawharu (2000), *kaitiakitanga* served as a means to ensure both the survival and availability of resources. Those who are referred to as *Kaitiaki* are Guardians (also known as Chiefs, Elders, and ritual specialists) who hold rights to their ancestral territories and are expected to maintain, protect, and restore the integrity of those resources for future generations. Despite this recognition, often there is considerable room for interpretation, resulting in a lack of meaningful involvement of Maori in environmental management (Newman & Moller 2005). Furthermore, there a limited exploration of a formal Guardian-program in the literature.

In Australia, the reciprocal relationship between Indigenous Peoples and the land is encapsulated by the saying: “if you look after the country, the country will look after you” (Griffiths & Kinnane 2010; p. iii). While the term, *Caring for Country*, generally refers to the approach that Indigenous Peoples take to land and sea ‘management’ (ISLM) in Australia, the concept of is an inappropriate translation of the entirety of ‘country’ (Austin at el. 2018). For instance, Indigenous peoples in Bawaka country define *country* as “a word in Aboriginal English which includes not just the territorial, land-based notion of a home land, but encompasses humans as well as waters, seas and all that is tangible and non-tangible, and which become together in a mutually caring and multidirectional manner to create and nurture a homeland” (Bawaka Country et al. 2016; p. 186).

The concept *Caring for Country* has been well documented in the literature (refer to Young 1987 and Rose 1992 for early discussions), with three principle phases: i) an emphasis on the continuity and transformation between human and land relationships (Young 1987; Young et al. 1991); ii) a shift in emphasis to the specific expertise of conservation and land management (Nugent 1988; Rose 1995); and iii) a technocratic model co-opted by the state to describe Indigenous involvement in a wide range of policy areas (Altman & Whitehead 2003). This third phase is arguably where we are currently: the extensive formalization of *Caring for Country* programs (Mackie & Meacheam 2018). For instance, in the Northern Territory, the Northern Land Council formalized the first *Caring for Country* unit in 1995. This program – and the subsequently creation of the federal *Caring for Our Country* program – has been extensively reviewed with numerous benefits: health and well-being (Burgess et al. 2005); economic (Hunt 2010; Morrison 2007); environmental (Garnett & Sithole 2007); and cultural and socio-political benefits (Griffiths & Kinnane 2010; Hunt Altman & May 2009).

In viewing this – and a desire to invest in the economic outcomes of Indigenous Peoples – the federal government launched *Working on Country* officially in 2007 (Vemuir & Gorman 2010). With an initial investment of $47.6 million over 4 years, the program had two initial objectives: i) support Indigenous Peoples to undertake environmental work that simultaneously met their desire of Caring for Country, while also fulfilling some of Australia’s environmental responsibilities; and ii) provide a mechanism for Indigenous Peoples to move into quality jobs. (Mackie & Meacheam 2016). These economic benefits (i.e. the second objective) have been cited as the rationale for broad political support, “…increas[ing] the likelihood of Cabinet support” (Mackie & Meacheam 2016: p. 160). The support and uptake by Indigenous Peoples to create Ranger programs, has been further bolstered by an additional $320 million over 5 years to support up to 730 Indigenous rangers across the country (Australian Government, 2018). At the same time, the program was transitioned to the Prime Minister’s Office, solidifying the emphasis on Indigenous empowerment, rather than environmental protection (Mackie & Meacheam 2016).

### 2.2 Canada & United States of America

Canada and the United States have vastly different experiences with their Indigenous Peoples. There was limited literature on Indigenous Guardians, or similar nomenclature, in the United States. This does not signify that there are no Guardian-like programs in the US, but it signifies that they might not be i) federally supported; or ii) coordinated across jurisdictions. Similar to above, the history of Indigenous Peoples in the US is out of the scope of this paper, but Dunbar-Ortiz (2014) is a powerful resource to understand the history of genocide in the settling and creation of what we now know as the United States. In Canada, the history of Guardians differs from Australia in two ways: i) the history of federal intervention in the creation and support of Indigenous Guardians is very recent; and ii) there is no unifying concept, such as Caring for Country, for Indigenous land and sea management, rather each Indigenous Nation (and language group) may have a different term for living in harmony and balance with Mother Earth. Often, these reciprocal relationships with Mother Earth, the lands and waters, and all non-human beings have been operating since time immemorial (Borrows 2002). Despite a deliberate attempt by Canada to remove these relationships (refer to Alfred 2009; Coulthard 2014; Reed et al. *under review*), Nations are increasingly reasserting governance through efforts to monitor, enforce, and assert jurisdiction in ancestral territories. Recently, this has manifested in a growing number of Indigenous Nations using a Guardians program model – garnering interested among First Nations, Métis, and Inuit alike (Dehcho First Nations et al. 2016).

There are approximately 30 programs already in place across Canada, including the Haida Watchmen, the Innu Environmental Guardians, and the Lutsel K’e Ni Hat’Ni Dene. Another example, the Coastal Guardian Watchmen Network, has been run by First Nations on the North and Central Coast of Haida Gwaii since 2005 as a network of eight monitoring programs. These long-standing Guardian programs have largely been developed in ad-hoc partnerships with federal departments (Fisheries and Oceans and Parks Canada), universities, and other institutions; and funded through a combination of philanthropic, federal, and other grants. After much advocacy from *Indigenous Nations (such as the Assembly of First Nations resolution: Support* *for First Nations Guardians*) and the Indigenous Leadership Initiative, the federal government announced funding ($26 million over 4 years) for the *Indigenous Guardians Pilot Program*. To date, 28 programs were funded in Year 1 and 33 were funded in Year 2^[[1]](#footnote-1)^ .

While these programs have a variety of scope, scale, and objectives, the overarching goal is to have "moccasins on the ground" to track the effects of resource development, a changing climate, and industrial development (Parlee, Geertsema & Willier 2012; Kotaska 2013; Dehcho First Nations et al. 2016). Guardians are employed in Indigenous territories on full, part-time, year-round and/or seasonal contracts to do a wide variety of tasks, including: community-based environmental stewardship (Griffiths & Kinnane 2010); support cultural revitalization and intergenerational knowledge sharing (Peachey 2015); monitor activities on their lands and territories (Dehcho First Nations et al 2016); and support wildlife and harvest monitoring (Garnett & Sithole 2007); monitor ecological health, maintain cultural sites and protect sensitive areas and species within their traditional territories. These efforts have been found to foster community cohesion, self reliance, self confidence and a sense of purpose (Kral et al. 2011; Wexler et al. 2014), and access and connection to land based activities have also been shown to alleviate depression, anxiety and even suicidal tendencies in school-aged children (Macdonald et al. 2013).

**Literature Cited**

Alessa L, Kliskey A, Gamble J, Fidel M, Beaujean G, Gosz J. 2016. The role of Indigenous science and local knowledge in integrated observing systems: moving toward adaptive capacity indices and early warning systems. Sustainability Science **11**: 91–102.

Alfred T. 2009. Colonialism and State Dependency. Journal of Aboriginal Health **5:** 42–60.

Altman J, Whitehead PJ. 2003. Caring for country and sustainable indigenous development: opportunities, constraints and innovation. Centre for Aboriginal Economic Policy Research, Canberra, Australia.

Austin BJ, Robinson CJ, Fitzsimons JA, Sandford M, Ens EJ, Macdonald JM, … Garnett ST. 2018. Integrated Measures of Indigenous Land and Sea Management Effectiveness: Challenges and Opportunities for Improved Conservation Partnerships in Australia. Conservation & Society **16**: 372–384.

Austin BJ, Vigilante T, Cowell S, Dutton IM, Djanghara D, Mangolomara S, … Clement Z. 2017. The Uunguu Monitoring and Evaluation Committee: Intercultural Governance of a Land and Sea Management Programme in the Kimberley, Australia. Ecological Management & Restoration **18**:124–133.

Bawaka Country, Wright S, Suchet-Pearson S, Lloyd K, Burarrwanga L, Ganambarr R, Ganambarr-Stubbs M, Ganambarr B, Maymuru D, Sweeney J. 2016. Co-becoming Bawaka: Towards a relational understanding of place/space. Progress in Human Geography **40**: 455-475.

Borrows J. 2002. Recovering Canada: the resurgence of Indigenous Law. Toronto, ON, University of Toronto Press.

Burgess C, Johnston F, Bowman D, Whitehead P. 2005. ‘Healthy country: healthy people? Exploring the health benefits of Indigenous natural resource management’. Australian and New Zealand Journal of Public Health **29:** 117-122.

Coulthard G. 2014. Red skin, white masks: Rejecting the colonial politics of recognition. Minneapolis MN, University of Minnesota Press.

Dehcho First Nations, ILI (Indigenous Leadership Initiative), Tides Canada. 2016. *Analysis of the Current and Future Value of Indigenous Guardian Work in Canada’s Northwest Territories*. ILI, Yellowknife, NWT. Available from: <http://www.ilinationhood.ca/wp-content/uploads/2016/11/value-in-indigenous-guardian-work-nwt.pdf>

Dunbar-Ortiz R. 2014. An Indigenous Peoples' History of The United States. Boston, MA, Beacon Press.

Garnett S, Sithole B. 2007. Sustainable Northern Landscapes and the Nexus with Indigenous Health: Healthy Country, Healthy People. Land & Water Australia. Canberra, Aus. Available from: http://citeseerx.ist.psu.edu/viewdoc/download?doi=10.1.1.363.8351&rep=rep1&type=pdf

Gorman J, Vemuri S. (2010). Payment for ecosystem services (PES) as a model for integrated natural cultural resource management (INCRM). International Journal of Environmental, Cultural, Economic and Social Sustainability **6**: 11-21.

Griffiths S, Kinnane S. 2010. Kimberley Aboriginal Caring for Country Plan — Healthy country, healthy people. Halls Creek, WA: Kimberley Language Resource Centre.

Heaslip R. 2008. Monitoring salmon aquaculture waste: the contribution of First Nations’ rights, knowledge, and practices in British Columbia, Canada. Marine Policy **32**: 988–996.

Hill R, Grant C, George M, Robinson C.J, Jackson S, Abel N. 2012. A typology of Indigenous engagement in Australian environmental management: Implications for knowledge integration and social-ecological system sustainability. Ecology and Society **17:**23.

Hunt J, Altman J, May K. 2009. Social Benefits of Aboriginal Engagement in Natural Resource Management, CAEPR Working Paper No. 60/2009, Centre for Aboriginal Economic Policy Research, Australian National University, Canberra.

Hunt J. 2010. Looking After Country in New South Wales: Two Case Studies of Socioeconomic Benefits for Aboriginal People, CAEPR Working Paper No. 75/2010, Centre for Aboriginal Economic Policy Research, Australian National University, Canberra.

Kahui V. Richards AC. 2014. Lessons from resource management by indigenous Māori in New Zealand: Governing the ecosystems as a commons. Ecological Economics **102:** 1-7.

Kawharu M. 2000. Kaitiakitanga: a Māori anthropological perspective of the Māori socio-environmental ethic of resource management. The Journal of the Polynesian Society **109**: 349-370.

Kerins S. 2012. Caring for Country to Working on Country. (Pages 26-44) in J. Altman and S. Kerins, editors, Caring for Country to Working on Country. Sydney, NSW, The Federation Press.

Kotaska JG. 2013. Reconciliation 'at the end of the day': Decolonizing Territorial Governance in British Columbia after Delgamuukw (Doctoral dissertation, University of British Columbia). <https://doi.org/10.14288/1.0074235>

Kral MJ, Idlout L, Minore JB, Dyck RJ, Kirmayer LJ. 2011. Unikkaartuit: meanings of well-being, unhappiness, health, and community change among Inuit in Nunavut, Canada. American Journal of Community Psychology **48**: 426-438.

MacDonald JP, Ford JD, Cunsolo-Willox A, Ross NA. 2013. A review of protective factors and causal mechanisms that enhance the mental health of Indigenous Circumpolar youth. International Journal of Circumpolar Health **72**.

Mackie K, Meacheam D. 2016. Working on country: a case study of unusual environmental program success. Australasian Journal of Environmental Management **23**:157-174.

May K. 2010. Government support for Indigenous cultural and natural resource management in Australia: The role of the Working on Country program. Australian Journal of Social Issues **45**: 395–416.

Morad M. Jay M. 2000. Kaitiakitanga: protecting New Zealand's native biodiversity. Biologist **47:** 197-201.

Morrison J. 2007. Caring for country. Pages 249-262 in J. Altman, and M. Hinkson, *editors,* Coercive Reconciliation: Stabilise, normalise, exit Aboriginal Australia, Melbourne, VIC: Arena Publications.

Muller S. 2014. Co-motion: Making space to care for country. Geoforum **54**: 132–141.

Newman J. Moller H. 2005. Use of matauranga (Māori traditional knowledge) and science to guide a seabird harvest: getting the best of both worlds? Senri Ethnological Studies **67**: 303-321.

Nugent R. 1988. Aboriginal Attitudes to Feral Animals and Land Degradation. Central Land Council, Alice Springs, Australia.

Peachey K. 2015. On-The-Ground Indigenous Stewardship Programs Across Canada: Inventory Project. Tides Canada, Vancouver, BC. Available from https://www.indigenousguardianstoolkit.ca/sites/default/files/Community Resource_Final Report with Profiles March 27 2015_1.pdf

Parlee B, Goddard E, Łutsël K’é Dene First Nation, Smith M. 2014. Tracking change: Traditional knowledge and monitoring of wildlife health in northern Canada. Human Dimensions of Wildlife **19**(1): 47-61.

Reed G, Brunet NB, Natcher DC. (in press). Can Indigenous community-based monitoring act as a tool for sustainable self-determination? Extractive Industries and Society.

Rose B. 1995. Land Management Issues: Attitudes and Perceptions Amongst the Aboriginal People of Central Australia. Central Land Council, Alice Springs, Australia.

Rose D. 1992. Dingo Makes Us Human: Life and land in an Australian Aboriginal culture, Cambridge University Press, Cambridge, UK.

Wexler L, Joule L, Garoutte J, Mazziotti J, Hopper K. 2014. Being responsible, respectful, trying to keep the tradition alive: Cultural resilience and growing up in an Alaska Native community, Transcultural Psychiatry **51**.

Young E. 1987. Resettlement and caring for the country: The Anmatyerre experience. Aboriginal History **11**: 156–170.

Young EH, Ross H, Johnson J, Kesteven J. 1991. Caring for Country: Aborigines and Land Management Australian National Parks and Wildlife Service, Canberra, Australia.

1. For more information, please refer to: https://www.canada.ca/en/environment-climate-change/services/environmental-funding/indigenous-guardians-pilot-program/map.html [↑](#footnote-ref-1)
