# Supplementary material for: Indigenous guardians as an emerging approach to indigenous environmental governance
Source: Conserv Biol. 2020 Jul 17;35(1):179–89. doi: 10.1111/cobi.13532 (PMC7984387; doi:10.1111/cobi.13532)
Supplement: Supplementary file 2 — Supplementary Material [file COBI-35-179-s001.docx]

### Appendix S2: References (in alphabetical order)

* marks a text for full-text review (n=24)

*Ansell S, Koenig J. 2011. CyberTracker: an integral management tool used by rangers in the Djelk Indigenous Protected Area, central Arnhem Land, Australia. Ecological Management & Restoration **12**(1): 13-25.

Artelle KA, Stephenson J, Bragg C, Housty JA, Housty WG, Kawharu M, Turner NJ. 2018. Values-led management: the guidance of place-based values in environmental relationships of the past, present, and future. Ecology and Society **23**(3):35

*Austin BJ, Robinson CJ, Fitzsimons JA, Sandford M, Ens EJ, Macdonald JM, … Garnett ST. 2018. Integrated Measures of Indigenous Land and Sea Management Effectiveness: Challenges and Opportunities for Improved Conservation Partnerships in Australia. Conservation & Society **16**: 372–384.

*Austin BJ, Vigilante T, Cowell S, Dutton IM, Djanghara D, Mangolomara S, … Clement Z. 2017. The Uunguu Monitoring and Evaluation Committee: Intercultural Governance of a Land and Sea Management Programme in the Kimberley, Australia. Ecological Management & Restoration **18**:124–133.

*Bach TM, Larson BM. 2017. Speaking about weeds: indigenous elders' metaphors for invasive species and their management. Environmental Values **26**(5) 561-581.

*Bach TM, Kulk CA, Rangan H. 2019. From killing lists to healthy country: Aboriginal approaches to weed control in the Kimberley, Western Australia. Journal of Environmental Management **229**:182-192.

Berkes F, Berkes MK, Fast H. 2007. Collaborative integrated management in Canada’s North: the role of local and traditional knowledge and community-based monitoring. Coastal Management **35**: 143–162.

Carmichael B. 2016. Supporting Indigenous rangers’ management of climate-change impacts on heritage sites: developing an effective planning tool and assessing its value. The Rangeland Journal **37**(6): 597-607.

Carmichael B, Wilson G, Namarnyilk I, Nadji S, Brockwell S, Webb B, Hunter F, Bird D. 2018. Local and Indigenous management of climate change risks to archaeological sites. Mitigation and adaptation strategies for global change **23**(2): 231-255.

Carmichael B, Wilson G, Namarnyilk I, Nadji S, Cahill J, Bird D. 2017. Testing the scoping phase of a bottom-up planning guide designed to support Australian Indigenous rangers manage the impacts of climate change on cultural heritage sites. Local Environment **22**(10): 1197-1216.

Carr A. 2002. Community science: bringing together social and scientific goals, Western Australia's Waterwatchers nurture both nature and neighbourhood.(Saving Place). Alternatives Journal 28(3): 29-31.

*Carroll C. 2014. Native enclosures: Tribal national parks and the progressive politics of environmental stewardship in Indian Country. Geoforum **53:** 31-40.

Carter JL, Hill GJ. 2007. Critiquing environmental management in indigenous Australia: two case studies. Area **39**(1): 43-54.

Clogg J, Askew H, Kung E, Smith G. 2016. Indigenous legal traditions and the future of environmental governance in Canada. Journal of Environmental Law and Practice **29**: 227-256.

Davies J, Hill R, Walsh FJ, Sandford M, Smyth D, Holmes MC. 2013. Innovation in management plans for community conserved areas: experiences from Australian indigenous protected areas. Ecology and Society **18**(2).

Davies J, Walker J, Maru YT. 2018. Warlpiri experiences highlight challenges and opportunities for gender equity in Indigenous conservation management in arid Australia. Journal of Arid Environments **149**: 40-52.

Dobbs RJ, Davies CL, Walker ML, Pettit NE, Pusey BJ, Close PG, Akune Y, Walsham N, Smith B, Wiggan A, Cox P. 2016. Collaborative research partnerships inform monitoring and management of aquatic ecosystems by Indigenous rangers. Reviews in Fish Biology and Fisheries **26**(4): 711-725

Ens EJ, Cooke P, Nadjamerrek R, Namundja S, Garlngarr V, Yibarbuk D. 2010. Combining Aboriginal and non-Aboriginal knowledge to assess and manage feral water buffalo impacts on perennial freshwater springs of the Aboriginal-owned Arnhem Plateau, Australia. Environmental Management **45**(4): 751-758.

Ens EJ, Finlayson M, Preuss K, Jackson S, Holcombe S. 2012. Australian approaches for managing ‘country’ using Indigenous and non‐Indigenous knowledge. Ecological Management & Restoration **13**(1): 100-107.

*Ens EJ, Scott ML, Yugul Mangi Rangers, Moritz C, Pirzl R. 2016. Putting indigenous conservation policy into practice delivers biodiversity and cultural benefits. Biodiversity and Conservation **25**(14): 2889-2906.

*Ens EJ, Towler GM, Daniels C, Yugul Mangi Rangers, Manwurrk Rangers. 2012. Looking back to move forward: collaborative ecological monitoring in remote Arnhem Land. Ecological Management & Restoration **13**(1): 26-35.

*Fache E. 2014. Caring for Country, a Form of bureaucratic participation. Conservation, development, and neoliberalism in Indigenous Australia. Anthropological Forum **24:** 267-286

*Fache E, Moizo B. 2015. Do burning practices contribute to caring for country? Contemporary uses of fire for conservation purposes in Indigenous Australia. Journal of Ethnobiology **35:** 163-182.

Fitzsimons J, Russell‐Smith J, James G, Vigilante T, Lipsett‐Moore G, Morrison J, Looker M. 2012. Insights into the biodiversity and social benchmarking components of the Northern Australian fire management and carbon abatement programmes. Ecological Management & Restoration **13**(1): 51-57.

Franklin DC, Petty AM, Williamson GJ, Brook BW, Bowman DM. 2008. Monitoring contrasting land management in the savanna landscapes of northern Australia. Environmental Management **41**(4): 501-515.

Gauvreau AM, Lepofsky D, Rutherford M, Reid M. 2017. “Everything revolves around the herring” the Heiltsuk–herring relationship through time. Ecology and Society **22**(2).

Gearheard S, Aporta C, Aipellee G, O’Keefe K. 2011. The Igliniit project: Inuit hunters document life on the trail to map and monitor arctic change. The Canadian Geographer/Le Géographe canadien **55**(1): 42-55.

Gill H. Lantz T. 2014. A community-based approach to mapping Gwich'in observations of environmental changes in the Lower Peel River Watershed, NT. Journal of Ethnobiology **34**(3): 294-314.

Gérin-Lajoie J, Herrmann TM, MacMillan GA, Hébert-Houle É, Monfett M, Rowell JA, Anaviapik Soucie T, Snowball H, Townley E, Lévesque E, Amyot M, Franssen J, Dedieu JP. 2018. IMALIRIJIIT: a community-based environmental monitoring program in the George River watershed, Nunavik, Canada. Écoscience **25**(4): 381-399.

*Gorman J, Vemuri S. 2012. Social implications of bridging the gap through ‘caring for country’ in remote Indigenous communities of the Northern Territory, Australia. The Rangeland Journal **34**(1): 63-73.

Grech A, Parra GJ, Beasley I, Bradley J, Johnson S, Whiting S, Marsh H. 2014. Local assessments of marine mammals in cross-cultural environments. Biodiversity and Conservation**23**(13): 3319-3338.

Greiner R. 2010. Payments for environmental services (PES): contribution to Indigenous livelihoods. WIT Transactions on Ecology and the Environment **131:** 163-174.

Gunn R, Hardesty BD, Butler J. 2010. Tackling ‘ghost nets’: local solutions to a global issue in northern Australia. Ecological Management & Restoration **11**(2): 88-98.

Harmsworth G, Awatere S, Robb M. 2016. Indigenous Māori values and perspectives to inform freshwater management in Aotearoa-New Zealand. Ecology and Society **21**(4).

Haynes C. 2017. The value of work and ‘common discourse’ in the joint management of Kakadu National Park. The Australian Journal of Anthropology **28**(1): 72-87.

Hepi M, Foote J, Makey L, Badham M, Te Huna A. 2018. Enabling mātauranga-informed management of the Kaipara Harbour, Aotearoa New Zealand. New Zealand journal of marine and freshwater research **52**(4): 497-510

Holmes MC, Jampijinpa W. 2013. Law for country: The structure of Warlpiri ecological knowledge and its application to natural resource management and ecosystem stewardship. Ecology and Society **18:**3.

Housty WG, Noson A, Scoville GW, Boulanger J, Jeo RM, Darimont CT, Filardi CE. 2014. Grizzly bear monitoring by the Heiltsuk people as a crucible for First Nation conservation practice. Ecology and Society **19**(2).

*Hunt J. 2012. ‘Caring for country’: a review of Aboriginal engagement in environmental management in New South Wales. Australasian Journal of Environmental Management **19**(4): 213-226.

Izurieta A, Sithole B, Stacey N, Hunter-Xenie H, Campbell B, Donohoe P, Brown J, Wilson L. 2011. Developing indicators for monitoring and evaluating joint management effectiveness in protected areas in the Northern Territory, Australia. Ecology and Society **16**(3): 9.

Jackson MV, Kennett R, Bayliss P, Warren R, Waina N, Adams J, Cheinmora L, Vigilante T, Jungine E, Woolagoodja K. Woolagoodja F. 2015. Developing collaborative marine turtle monitoring in the Kimberley region of northern Australia. Ecological Management & Restoration **16**(3): 163-176.

Kahui V, Richards AC. 2014. Lessons from resource management by indigenous Māori in New Zealand: Governing the ecosystems as a commons. Ecological Economics **102:** 1-7.

Kawharu M. 2000. Kaitiakitanga: a Māori anthropological perspective of the Māori socio-environmental ethic of resource management. The Journal of the Polynesian Society **109**: 349-370.

Kennett R, Munungurritj N, Yunupingu D. 2004. Migration patterns of marine turtles in the Gulf of Carpentaria, northern Australia: implications for Aboriginal management. Wildlife Research **31**(3): 241-248.

Lagasse CR, Ou W, Honka LD, Atlas WI, Hutton CN, Kotaska J, Hocking MD. 2014. Design considerations for community-based stream monitoring to detect changes in Pacific salmon habitats. Ecology and Society **19**(4).

Lemelin H, Maher P. 2009. Nanuk of the Torngats: human–polar bear interactions in the Torngat mountains National Park, Newfoundland and Labrador, Canada. Human Dimensions of Wildlife **14**(2):152-155.

Leonard S, Parsons M, Olawsky K, Kofod F. 2013. The role of culture and traditional knowledge in climate change adaptation: Insights from East Kimberley, Australia. Global Environmental Change **23**(3): 623-632.

Lyver POB. 2005. Co-managing environmental research: lessons from two cross-cultural research partnerships in New Zealand. Environmental Conservation**32**(4): 365-370.

Lyver POB, Richardson SJ, Gormley AM, Timoti P, Jones CJ, Tahi BL. 2018. Complementarity of indigenous and western scientific approaches for monitoring forest state. Ecological Applications **28**(7): 1909-1923.

Lyver POB, Timoti P, Jones CJ, Richardson SJ, Tahi BL, Greenhalgh S. 2017. An indigenous community-based monitoring system for assessing forest health in New Zealand. Biodiversity and Conservation **26**(13): 3183-3212.

*Mackie K, Meacheam D. 2016. Working on country: a case study of unusual environmental program success. Australasian Journal of Environmental Management **23**:157-174.

*McMillan LJ, Prosper K. 2016. Remobilizing netukulimk: indigenous cultural and spiritual connections with resource stewardship and fisheries management in Atlantic Canada. Reviews in fish biology and fisheries **26**(4): 629-647.

Moewaka Barnes H, Eich E, Yessilth S. 2019. Colonisation, hauora and whenua in Aotearoa. Continuum: Journal of the Royal Society of New Zealand **49:** 19-33.

Morad M, Jay M. 2000. Kaitiakitanga: protecting New Zealand's native biodiversity. Biologist **47:** 197-201.

*Muller S. 2008. Indigenous payment for environmental service (PES) opportunities in the Northern Territory: negotiating with customs. Australian geographer **39**(2): 149-170.

Muller S. 2014. Co-motion: Making space to care for country. Geoforum **54**: 132–141.

Muller S, Power ER, Suchet-Pearson S, Wright S, Lloyd K. 2009. “Quarantine matters!”: quotidian relationships around quarantine in Australia's northern borderlands. Environment and Planning A **41**(4): 780-795.

Noble M, Duncan P, Perry D, Prosper K, Rose D, Schnierer S, Tipa G, Williams E, Woods R, Pittock J. 2016. Culturally significant fisheries: keystones for management of freshwater social-ecological systems. Ecology and Society **21**(2).

Parlee B, Goddard E, Łutsël K’é Dene First Nation, Smith M. 2014. Tracking change: Traditional knowledge and monitoring of wildlife health in northern Canada. Human Dimensions of Wildlife **19**(1): 47-61.

*Perry JJ, Sinclair M, Wikmunea H, Wolmby S, Martin D, Martin B. 2018. The divergence of traditional Aboriginal and contemporary fire management practices on Wik traditional lands, Cape York Peninsula, Northern Australia. Ecological Management & Restoration **19**(1): 24-31.

Polfus JL, Manseau M, Simmons D, Neyelle M, Bayha W, Andrew F, Andrew L, Klütsch CF, Rice K, Wilson P. 2016. Łeghágots' enetę (learning together) the importance of indigenous perspectives in the identification of biological variation. Ecology and Society **21**(2).

Prangnell J, Ross A, Coghill B. 2010. Power relations and community involvement in landscape‐based cultural heritage management practice: an Australian case study. International Journal of Heritage Studies **16**(1-2): 140-155.

*Preuss K, Dixon M. 2012. ‘Looking after country two-ways’: Insights into Indigenous community-based conservation from the Southern Tanami. Ecological Management & Restoration **13**:2-15

*Pyke M, Toussaint S, Close P, Dobbs R, Davey I, George K, Oades D, Sibosado D, McCarthy P, Tigan C, Riley E. 2018. Wetlands need people: a framework for understanding and promoting Australian indigenous wetland management. Ecology and Society **23**

Roberts M, Norman W, Minhinnick N, Wihongi D, Kirkwood C.1995. Kaitiakitanga: Māori perspectives on conservation. Pacific Conservation Biology **2**(1): 7-20.

*Robinson CJ, Smyth D, Whitehead PJ. 2005. Bush tucker, bush pets, and bush threats: cooperative management of feral animals in Australia's Kakadu National Park. Conservation Biology **19**(5): 1385-1391.

Robinson CJ, Wallington TJ. 2012. Boundary work: engaging knowledge systems in co-management of feral animals on Indigenous lands. Ecology and Society **17**(2).

Sherman KP, Van Lanen J, Sherman RT. 2010. Practical environmentalism on the Pine Ridge Reservation: Confronting structural constraints to Indigenous stewardship. Human Ecology **38**: 507-520.

Spencer‐Cotton A, Kragt ME, Burton M. 2018. Spatial and scope effects: valuations of coastal management practices. Journal of Agricultural Economics **69**(3): 833-851.

*Vemuri S, Gorman J. 2010. Enhancing natural resource management through payment for ecosystem services. WIT Transactions on Ecology and the Environment **131**: 175-186.

Vigilante T, Ondei S, Goonack C, Williams D, Young P, Bowman DM. 2017. Collaborative research on the ecology and management of the ‘Wulo’monsoon rainforest in Wunambal Gaambera Country, North Kimberley, Australia. Land**6**(4): 68.

Waltham NJ, Schaffer J, Buist J, Geyle M, Toby D. 2018. Working with land and sea rangers to tackle tropical wetland restoration and conservation on the north-western islands, Torres Straits, Australia. Wetlands ecology and management **26**(6): 1143-1156.

Weston N, Bramley C, Bar‐Lev J, Guyula M, O’Ryan S. 2012. Arafura three: Aboriginal ranger groups protecting and managing an internationally significant swamp. Ecological Management & Restoration **13**(1): 84-88.

Whitehouse H, Watkin Lui F, Sellwood J, Barrett MJ, Chigeza P. 2014. Sea country: Navigating Indigenous and colonial ontologies in Australian environmental education. Environmental Education Research **20**(1): 56-69.

Wilson NJ. 2014. Indigenous water governance: Insights from the hydrosocial relations of the Koyukon Athabascan village of Ruby, Alaska. Geoforum **57**:1-11.

*Wilson NJ, Mutter E, Inkster J, Satterfield T. 2018. Community-based monitoring as the practice of Indigenous governance: a case study of Indigenous-led water quality monitoring in the Yukon River Basin. Journal of Environmental Management **210**: 290–298

*Wiseman ND, Bardsley DK. 2016. Monitoring to Learn, Learning to Monitor: A Critical Analysis of Opportunities for Indigenous Community‐Based Monitoring of Environmental Change in Australian Rangelands. Geographical Research **54**(1): 52-71.

*Woodward E. 2008. Social networking for Aboriginal land management in remote northern Australia. Australasian Journal of Environmental Management **15**: 241-252.

Wyatt S, Hébert M, Fortier JF, Blanchet ÉJ, Lewis N. 2019. Strategic approaches to Indigenous engagement in natural resource management: use of collaboration and conflict to expand negotiating space by three Indigenous nations in Quebec, Canada. Canadian Journal of Forest Research **49**(4): 375-386.

Zander KK, Austin BJ, Garnett ST. 2014. Indigenous peoples’ interest in wildlife-based enterprises in the Northern Territory, Australia. Human Ecology **42**(1): 115-126.

Zander KK, Dunnett DR, Brown C, Campion O, Garnett ST. 2013. Rewards for providing environmental services—Where indigenous Australians' and western perspectives collide. Ecological Economics **87**: 145-154.

Zurba M, Berkes F. 2014. Caring for country through participatory art: creating a boundary object for communicating Indigenous knowledge and values. Local Environment **19**(8): 821-836.

*Zurba M, Ross H, Izurieta A, Rist P, Bock E, Berkes F. 2012. Building co-management as a process: problem solving through partnerships in Aboriginal country, Australia. Environmental Management **49**(6): 1130-1142.
