# Supplementary material for: Indigenous guardians as an emerging approach to indigenous environmental governance
Source: Conserv Biol. 2020 Jul 17;35(1):179–89. doi: 10.1111/cobi.13532 (PMC7984387; doi:10.1111/cobi.13532)
Supplement: Supplementary file 3 — Supplementary Material [file COBI-35-179-s003.docx]

### **Appendix S3: Biographical notes about the authors**

Graeme Reed is a PhD candidate at the University of Guelph, studying the intersection of Indigenous governance, environmental governance and the climate crisis. At the same time, he works at the Assembly of First Nations (AFN) as a Senior Policy Advisor in the Environment Sector. He is of mixed Anishinaabe and European descent.

Nicolas D. Brunet is the Latornell Professor in Environmental Stewardship at the University of Guelph with an active research program in the community-based conservation field coupled with large scale social-ecological mixed method studies at international and national scales. His research primarily focuses upon natural resource governance and the tools, such as Indigenous community-based monitoring and community science, used to measure the impacts of resource extraction in boreal and arctic ecosystems.

Sheri Longboat is an Associate Professor at the University of Guelph and member of the Six Nations of the Grand River. Her research focuses on issues of water insecurity, the relationship with natural resources, and other environmental challenges through the development of solutions that integrate science, policy and the knowledge and experiences of Indigenous Peoples

David Natcher is a Professor in the Department of Agricultural and Resource Economics at the University of Saskatchewan. Dr. Natcher conducts research in the areas of environmental and economic anthropology. He currently serves as the Canadian representative on both the International Arctic Science Committee's Social and Human Working Group and the Arctic Council's Social, Economic and Cultural Expert Group.
